# Revealing a causal relationship between gut microbiota and lung cancer: a Mendelian randomization study

**DOI:** 10.3389/fcimb.2023.1200299

**Published:** 2023-09-27

**Authors:** Yingchen Li, Ke Wang, Yuchong Zhang, Jitao Yang, Ying Wu, Mingfang Zhao

**Affiliations:** ^1^ Department of Medical Oncology, The First Hospital of China Medical University, Shenyang, China; ^2^ Department of Urology, The First Affiliated Hospital of China Medical University, Shenyang, China; ^3^ Phase I Clinical Trails Center, The First Hospital of China Medical University, Shenyang, China

**Keywords:** lung cancer, gut microbiota, Mendelian randomization, causal inference, genome-wide association study

## Abstract

**Background:**

The gut microbiota has been found to be associated with the risk of lung cancer. However, its causal relationship with various types of lung cancer remains unclear.

**Methods:**

We conducted a Mendelian randomization (MR) study using the largest genome-wide association analysis of gut microbiota data to date from the MiBioGen consortium, with pooled statistics for various types of lung cancer from the Transdisciplinary Research in Cancer of the Lung, the International Lung Cancer Consortium, and FinnGen Consortium R7 release data. Inverse variance weighted, weighted model, MR-Egger regression, and weighted median were adapted to assess the causal relationship between gut microbiota and various types of lung cancer. Sensitivity analysis was used to test for the presence of pleiotropy and heterogeneity in instrumental variables. A reverse MR analysis was performed on these bacteria to determine their potential role in causing lung cancer. A reverse MR analysis was performed on these bacteria to determine their potential role in causing lung cancer. Multivariable Mendelian randomization (MVMR) was conducted to assess the direct causal impact of gut microbiota on the risk of various types of lung cancer.

**Results:**

Using IVW as the primary analytical method, we identified a total of 40 groups of gut microbiota with potential causal associations with various subtypes of lung cancer, of which 10 were associated with lung cancer, 10 with lung adenocarcinoma, 9 with squamous cell lung cancer, and 11 groups of bacteria associated with small cell lung cancer. After performing FDR correction, we further found that there was still a significant causal relationship between Peptococcaceae and lung adenocarcinoma. Sensitivity analyses demonstrated the robustness of these results, with no heterogeneity or pleiotropy found.

**Conclusions:**

Our results confirm a causal relationship between specific gut microbiota and lung cancer, providing new insights into the role of gut microbiota in mediating the development of lung cancer.

## 1 Introduction

According to statistics, lung cancer accounts for 2.2 million new cases and 1.79 million fatalities each year (Thai et al., 2021), making it the second most commonly occurring cancer type globally and the primary cause of cancer-related deaths (Sung et al., 2021; Huang et al., 2022). Several well-known risk factors contribute to the development of lung cancer, including smoking, previous lung diseases, air pollutants, and occupational carcinogens (Samet et al., 2009; Wang et al., 2020). Identifying and discovering potentially modifiable risk factors is crucial to reducing the incidence of lung cancer, which is conducive to early diagnosis and treatment of lung cancer.

Recently, it has been suggested that the gut microbiota (GM) has an impact on the progression of lung cancer. GM refers to the highly complex community of microorganisms that reside within the gastrointestinal tract, including archaea, bacteria, eukaryotes, parasites, and viruses (Ge et al., 2021). Dysbiosis of microbial ecology may lead to changes in metabolism, immunosuppression, and the recruitment of inflammatory factors that can drive lung carcinogenesis. In the study by JinC and LagoudasGK et al., mouse models showed that the local microbiota associated with tumor development promotes inflammatory progression through lung-resident γδ T cells (Jin et al., 2019) and is thought to be associated with lung cancer. The most recent theory holds that even though the gastrointestinal tract and respiratory tract are physically separate, they share an embryonic origin and a high degree of structural similarity (Georgiou et al., 2021). There is a distinct crosstalk between the respiratory tract and the gastrointestinal tract known as the gut–lung axis (Dang and Marsland, 2019; Enaud et al., 2020; Zhang et al., 2020). The gut-lung axis is the pathway through which the GM interacts with the lung (Young et al., 2016; Liu et al., 2021), Microbiota-accessible carbohydrates can alter the GM and improve short-chain fatty acid (SCFA) levels, hence shaping lung immunity (Arrieta et al., 2015; Budden et al., 2017). Through T cell receptor signaling, SCFAs may activate innate lymphoid cells that produce IL-22 type 3, regulatory T cells, and Th2 cells in the lung to reduce inflammation and thus reduce the incidence of lung cancer (Corrêa et al., 2022). SCFAs can also exert effective anti-inflammatory and immunomodulatory effects by activating G protein-coupled cell surface receptors and inhibiting histone deacetylases, as well as exert immunomodulatory effects by activating G protein-coupled cell surface receptors and inhibiting histone deacetylases (Ubachs et al., 2021). Nonetheless, whether there is a clear causal relationship between lung cancer and GM still needs further proof.

In order to investigate the potential causal link between lung cancer and GM, Mendelian randomization (MR) analysis was used. MR analysis is a statistical strategy based on genome-wide association analysis, following the Mendelian rule of “parental alleles randomly assigned to offspring”. It is therefore a natural randomized controlled trial (RCT). MR analysis has been extensively utilized to investigate the connection between GM and diseases such as pre-eclampsia and eclampsia (Li et al., 2022), autoimmune diseases (Xu et al., 2021), and psychiatric disorders (Zhuang et al., 2020). In this research, we employed the two-sample MR analysis method to examine the potential impact of GM in various types of lung cancer. The findings of this research can serve as a foundation for understanding the etiology and diagnosis of lung cancer.

## 2 Materials and methods

### 2.1 Study design

**Figure 1:**
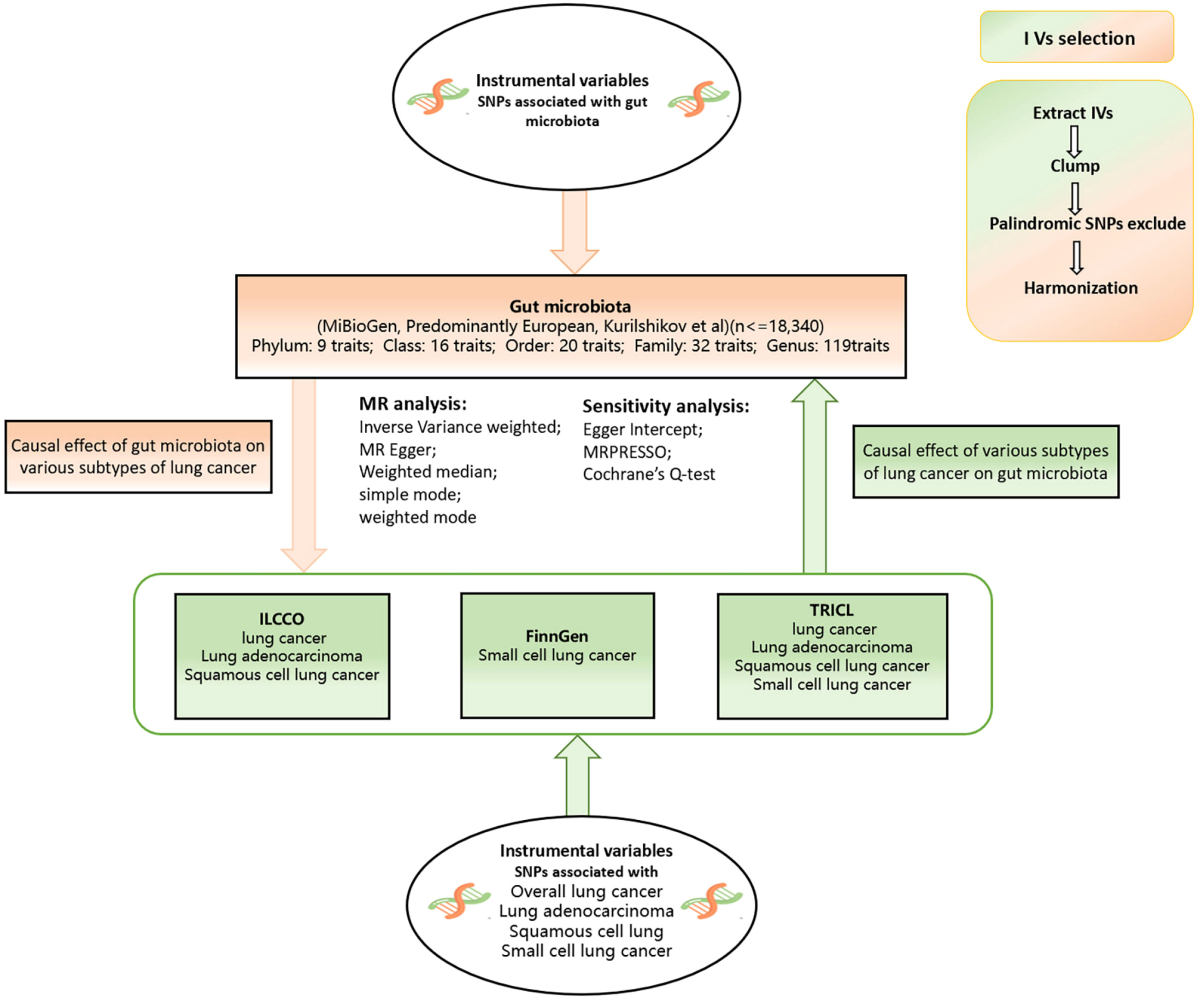
Flow chart of our study design. This study was a bidirectional Mendelian randomization analysis testing the causal effects between the gut microbiota and different types of lung cancer. Inverse variance weighted (IVW) was adopted as the primary method for univariable MR. To provide robust evidence of MR estimates, sensitivity analyses were used.

A bidirectional two-sample MR analysis was conducted to investigate the causal relationship between GM and various subtypes of lung cancer. The flow chart of the study is shown in **Figure 1**. The aim of reverse MR analysis is to mitigate the potential effect of various subtypes of lung cancer on the GM and improve the confidence of the results.

### 2.2 Data sources

The MiBioGen study provides genome-wide association summary data for the GM (Kurilshikov et al., 2023) and is the largest meta-analysis of its kind to date. The study includes 24 cohorts comprising 18,340 participants of various races and ages. To ensure data quality, the majority of cohorts employed comparable methods for interpolation and subsequent filtering. Direct taxonomic binning was used to classify the taxonomy, resulting in 211 taxa being included in the analysis, representing 131 genera, 35 families, 20 orders, 16 classes, and 9 phyla.

To bolster statistical power, validate research findings, and foster the exploration of potential new associations, we leveraged a comprehensive set of three lung cancer datasets. The Transdisciplinary Research in Cancer of the Lung (TRICL) is a member of an organization focused on Genetic Associations and Mechanisms in Oncology (GAME-ON consortium). The International Lung Cancer Consortium (ILCCO) is an international organization of lung cancer researchers that was founded in 2004 to share data from ongoing lung cancer epidemiological studies with the overall goal of maximizing statistical power. We obtained summary data from the MR-Base database for both TRICL and ILCCO. The FinnGen consortium R7 (http://r7.finngen.fi/) results provided us with summary data on small-cell lung cancer. All the data is listed in **Table 1**. We used all summary data from published studies and publicly available GWAS abstracts, and therefore did not require additional ethical approval or consent.

**Table 1:** The data source of exposure, outcome, and multivariate Mendelian randomization.

Overview of the source of gut microbiota.							
Traits	Sample size	Consortium	Population	download URL			
Gut microbiota	18340	MiBioGen	mixed	https://mibiogen.gcc.rug.nl/			
Overview of the source of various subtypes of lung cancer.							
GWAS ID	Trait	Consortium	Sample size	Case	Control	Number of SNPs	Population
ieu-a-987	Lung cancer	TRICL	85,449	29,863	55,586	10,439,018	European
ieu-a-984	Lung adenocarcinoma	TRICL	65,864	11,245	54,619	10,345,176	European
ieu-a-989	Squamous cell lung cancer	TRICL	62,467	7,704	54,763	10,341,529	European
ieu-a-988	Small cell lung carcinoma	TRICL	23,371	2,791	20,580	7,438,318	European
ieu-a-966	Lung cancer	ILCCO	27,209	11,348	15,861	8,945,893	European
ieu-a-965	Lung adenocarcinoma	ILCCO	18,336	3,442	14,894	8,881,354	European
ieu-a-967	Squamous cell lung cancer	ILCCO	18,313	3,275	15,038	8,893,750	European
finn-b-C3_SCLC	Small cell lung carcinoma	NA	218,792	179	218,613	16,380,466	European
Multivariate Mendelian randomization							
GWAS ID	Trait	Consortium	Sample size	Case	Control	Number of SNPs	Population
finn-b-J10_COPD	COPD	NA	447,485	6915	186723	16,380,382	European
ieu-a-1283	Alcohol consumption	UK Biobank	112,117	NA	NA	12,935,395	European
ieu-b-142	Cigarettes smoked per day	GSCAN	249,752	NA	NA	12,003,613	European

NA, Not Applicable.

### 2.3 Instrumental variable selection

For the MR analysis, we examined 211 taxa of gut microbes, but excluded 15 taxa from unclassified groups, leaving 196 bacterial taxa (9 phyla, 16 classes, 20 orders, 32 families, and 119 genera) to be included in the analysis. To ensure data robustness and reliability of results, quality control of SNPs was performed to obtain compliant instrumental variables:(1) After consulting the relevant research (Lv et al., 2021; Liu et al., 2022; Ren et al., 2023; Su et al., 2023), we adjusted the threshold to the locus-wide significance level (p < 1 × 10−5); (2) As strong linkage disequilibrium may cause biased results, we performed linkage disequilibrium (LD) analysis with a threshold of r2< 0.001 and an aggregation window of 10,000kb, using a reference panel of 1000 Genome Project European samples; (3) Exclusion of palindromic sequences and SNPs with allelic inconsistencies between the two samples; and (4) To evaluate the strength of the SNP, we calculated the F-statistic. An F-statistic value of ≥10 indicates a lack of strong evidence for weak instrument bias. We excluded weak instruments with F-statistics less than <10.

### 2.4 Statistical analysis

#### 2.4.1 MR analysis

To identify the connection between the GM and various types of lung cancer, we utilized four approaches in this study: inverse variance weighted (IVW), MR-Egger, weighted model, and weighted median. IVW is a method for MR to meta-summarize wald estimates for each locus when analyzing multiple SNPs. The IVW results would be objective if horizontal pleiotropy did not exist (Pierce and Burgess, 2013). The MR-Egger method employs the InSIDE assumption to perform a weighted linear regression of exposure outcomes, while the weighted median method assigns greater weight to SNPs with larger beta values during estimation. Weighted median is the median of the distribution function obtained by sorting all individual SNP effect values by weight. To obtain robust estimates in our research, we require at least 50% of the information to be derived from valid instrumental variables. We performed validation using the Benjamini-Hochberg correction. Only significant data with a corrected p-value of less than 0.05 were considered to have a strong causal relationship. However, we suggest that bacteria with a p-value of less than 0.05 but not significant after correction may still have a potential causal relationship with lung cancer. Furthermore, we conducted additional reverse MR analyses to investigate whether different types of lung cancer have a causal impact on the significant GM identified in our study. In order to investigate the influence of the GM on different subtypes of lung cancer, we conducted a multivariate Mendelian randomization analysis. This analytical approach allowed us to explore the causal relationship between the GM and lung cancer subtypes while controlling for potential confounding factors such as smoking, alcohol consumption, and chronic obstructive pulmonary disease (COPD).

#### 2.4.2 Sensitivity analysis

We utilized MR-PRESSO to evaluate horizontal pleiotropy, while MR-Egger avoided enforcing the regression line through zero to account for the existence of directional genetic pleiotropy. In cases where the regression intercepts were non-zero and p<0.05, we regarded them as statistically significant indicators of genetic pleiotropy. Furthermore, we conducted Cochran’s Q test, and we observed heterogeneity if p<0.05. Finally, we carried out a leave-one-out sensitivity test to determine if a single instrumental variable had a significant impact on the causal effect.

The R software was used to perform all statistical analyses (Version 4.2.2). “TwoSampleMR” package (version 0.5.6) (Hemani et al., 2017) and “MRPRESSO” (version 1.0) package (Verbanck et al., 2018) were used to perform MR analysis. The code used for our analysis is available on GitHub [GitHub].

## 3 Results

**Figure 2:**
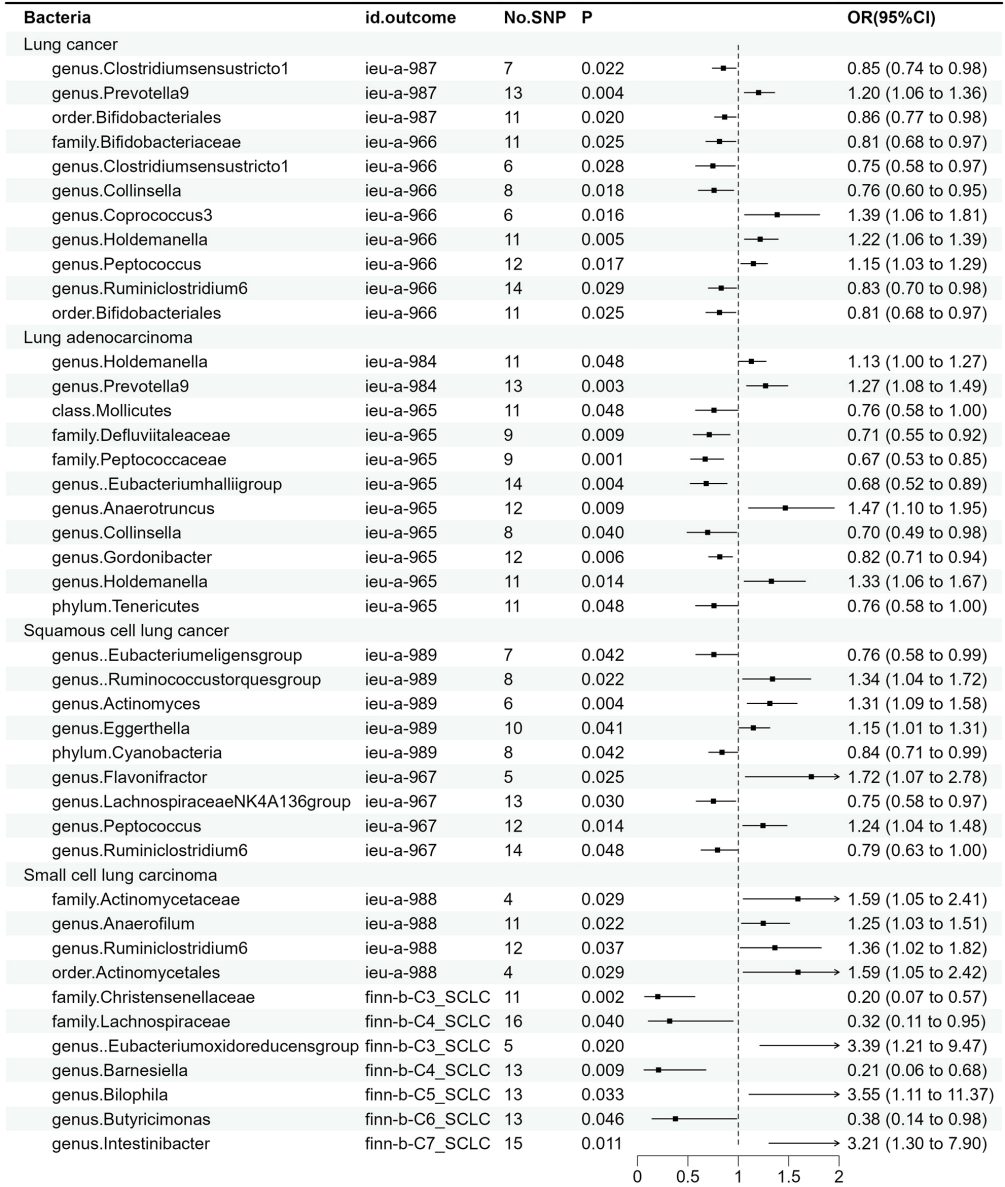
Associations between gut microbiota and various subtypes of lung cancer. The odds ratios are scaled per 1 standard deviation increase in gut microbiota.CI, confidence interval; OR, odds ratio.

The results of MR estimates for the relationship between the GM and different types of lung cancer are presented in **Supplementary Table 1** and **Figure 2**. After correction, one microbial taxon was found to be significant at a significance level of 0.05.

### 3.1 Causal effect of GM on various subtypes of lung cancer

For overall lung cancer, as shown in **Supplementary Figure 1**, we identified a total of 11 causal relationships between the GM and lung cancer in the ILCCO and TRICL datasets. Among these 11 microbial communities, Prevotella9 (P = 0.004), Coprococcus3 (P = 0.016), Holdemanella (P = 0.005), and Peptococcus (P = 0.017) were considered positively correlated with the risk of lung cancer, while Bifidobacteriaceae (P = 0.025), Collinsella (P = 0.018), and Ruminiclostridium6 were (P =0.029) deemed negatively correlated with lung cancer risk. Clostridiumsensustricto1 (P_1 = _0.022, P_2 = _0.028) and “Bifidobacteriales” (P_1 = _0.02, P_2 = _0.025) both exhibited positive results in two lung cancer datasets. Notably, “family Bifidobacteriaceae” and “order Bifidobacteriales” demonstrated a consistent effect in the same lung cancer dataset (P = 0.025).

For lung adenocarcinoma, as shown in **Supplementary Figure 2**, we identified a total of 11 causal relationships between the GM and lung adenocarcinoma in two datasets, among which 7 demonstrated an inversely proportional relationship with the risk of lung adenocarcinoma. To be specific, these encompass Mollicutes (p = 0.048), Defluviitaleaceae (p = 0.009), Peptococcaceae (p =0.001), Eubacteriumhalliigroup (p = 0.004), Collinsella (p = 0.04), Gordonibacter (p=0.06), and Tenericutes (p= 0.048), possibly because Mollicutes represent the class level, while Tenericutes represent the phylum level, with the latter encompassing the former. This is why these two microbial taxa exhibited similar effects on lung adenocarcinoma. Conversely, Prevotella9 (p = 0.003) and Anaerotruncus (p= 0.009) displayed a direct correlation with the risk of lung adenocarcinoma. It is worth noting that the Holdemanella has exhibited remarkable efficacy in both datasets. The Peptococcaceae was also found to be significant after P-value adjustment (p_adj = 0.04).

For squamous cell lung cancer, as demonstrated in **Supplementary Figure 3**, nine bacteria were identified by MR analysis as being associated with squamous cell lung cancer. Genetically predicted risk of squamous cell lung cancer was correlated with Ruminococcustorquesgroup (P = 0.022), Actinomyces (p = 0.004), Eggerthella (P = 0.041), Flavonifractor (P = 0.025), and Peptococcus (P = 0.014). The genetically predicted protective roles of microbiota are Eubacterium eligens (P = 0.042), Collinsella (p = 0.04), Lachnospiraceae (p = 0.03), and Ruminiclostridium6 (p = 0.048).

Furthermore, we also identified 11 bacteria associated with small cell lung cancer, as illustrated in **Supplementary Figure 4**. The results of IVW showed a causal correlation of Actinomycetaceae (P = 0.029), Actinomycetales (P = 0.029), Anaerofilum (P = 0.022), Ruminiclostridium6 (P = 0.037) Eubacterium oxidoreducens (P = 0.02), Bilophila (p = 0.033), and Intestinibacter (P = 0.011) on the risk of small cell lung cancer. Furthermore, the microbial communities Christensenellaceae (P = 0.002), Lachnospiraceae (P = 0.04), Barnesiella (P = 0.009), and Butyricimonas (P = 0.046) conferred a protective effect against small cell lung cancer.

### 3.2 Sensitivity analysis

Sensitivity analyses were performed to ensure the results’ robustness. The findings of Cochran’s Q showed that the instrumental variables were homogeneous. According to the results of the MR-Egger regression, no evidence of pleiotropic effect was observed (all P intercept >0.05), as well as MR-PRESSO global test (all P global test >0.05); this finding shows that IVs seem to be unlikely to influence lung cancer risk through pathways other than the GM with significant results. All results can be found in the **Supplementary File**. Furthermore, **Supplementary Figures 5**-**8** summarize the results of the causal effect of significant taxa on various subtypes of lung cancer in the leave-one-out analysis.

### 3.3 Reverse MR analysis

A reverse MR analysis was carried out to investigate whether different types of lung cancer have any causal impact on the observed significant bacteria. The reverse MR analysis process was identical to the former MR analysis. The majority of results indicated that there is no reverse causality (**Supplementary Tables 2**-**4**). Only the effect of small cell lung cancer on Holdemania exhibited a reverse causal relationship, and to ensure the rigor of our results, we excluded this finding.

### 3.4 MVMR analysis

To ascertain whether the significantly positive microbial community observed after calibration directly or indirectly affects cancer risk through common cancer risk factors, we performed additional MVMR analysis. Although multivariable Mendelian randomization (MR) has been used in some previous studies to consider the joint effects of multiple variables, we did not find that this approach was suitable for our current research. After considering important confounding factors such as smoking, alcohol consumption, and chronic obstructive pulmonary disease in our analysis, we were unable to identify any common SNPs across the majority of the GM, which limits the ability to establish a robust causal relationship using multivariable MR; the results are shown in **Supplementary Table 5**. Previous studies have shown that conducting multivariable MR analyses in the presence of insufficient common SNPs may lead to the accumulation of bias and errors in conclusions, lacking scientific justification (Sanderson, 2021). Only Peptococcus yielded common SNPs during the multivariable Mendelian randomization analysis; however, after adjustment, the results were no longer significant.

## 4 Discussion

In this study, we performed a two-sample MR analysis using the summary statistics of the GM from the MiBioGen consortium’s largest GWAS data and the summary statistics of various subtypes of lung cancer from the TRICL, ILCCO, and FinnGen consortium R7 release data to evaluate the causal association between GM and lung cancer. We found 10 GM to be causally associated with overall lung cancer, 10 with lung adenocarcinoma, 9 with squamous cell carcinoma, and 11 with small cell lung cancer. After FDR correction, we found Peptococcaceae to be causally associated with lung adenocarcinoma.

The GM primarily influences the occurrence of lung cancer *via* the gut-lung axis, exerting its effects by regulating the lung immune system, influencing lung inflammation response, and producing metabolites (Zhao et al., 2021). Alterations in the GM may lead to disruption of the intestinal mucosal barrier, resulting in the occurrence of inflammation. These inflammatory factors may enter the lungs through the gut-lung axis (Enaud et al., 2020; Zhao et al., 2021), and long-term inflammatory responses can promote the occurrence of lung cancer by activating signal transduction pathways. In addition, intestinal immune cells such as ILC2s, ILC3, and TH17 can migrate directly from the intestine to the respiratory tract *via* the bloodstream to influence the immune activity of the respiratory system (Ma et al., 2022). The GM can also produce various metabolites, such as SCFAs, carotenoids, and bile acids (Du et al., 2022; Soriano-Lerma et al., 2022). These metabolites may directly or indirectly affect the occurrence of lung cancer by entering the lungs through the gut-lung axis.

The family of Bifidobacteriaceae, as a probiotic, has been reported in a study to have a protective effect against oxidative stress-induced DNA damage *in vitro*. This study suggests that Bifidobacterium bifidum, a member of the Bifidobacteriaceae family, possesses antioxidant properties that may prevent diseases such as lung cancer by reducing DNA damage (Bhatt et al., 2017). Our research findings are consistent with the notion that Bifidobacteriaceae has a strong causal relationship with lung cancer and serves as a protective factor against it. With regard to cyanobacteria, phycocyanin can be produced, which has a variety of biological functions such as anti-tumor, anti-oxidant, immunomodulatory, and anti-inflammatory activities. Several studies have demonstrated that phycocyanin exerts a dual role in NSCLC cells by not only reducing the activity and proliferation of A549 cells (Hao et al., 2018a), but also inhibiting the proliferation of various NSCLC cell lines (such as H1299, H460, and LTEP-a2) while inducing apoptosis (Hao et al., 2018b). These findings provide a basis for future applications of cyanobacteria in the treatment of NSCLC. It is worth mentioning that our findings align with those of Nam et al., who found that patients with the inflammatory disease “rosacea” had lower levels of Peptococcaceae in the gut (Nam et al., 2018). Furthermore, chronic inflammation is known to be linked to lung cancer. Based on this, we propose that Peptococcaceae may play a role in mitigating lung inflammation damage and could potentially impact the development of lung cancer. However, there is a lack of relevant research evidence to confirm the specific mechanism of the potential causal link between these specific microbial taxa and the incidence of lung cancer.

The study by J et al. suggests that the dysbiosis of lung microbiota in lung cancer patients is associated with upregulation of the PI3K signaling pathway and cancer progression. Specific lung cancer-associated bacteria, such as Prevotella, may impact the tumor microenvironment and initiate a cascade reaction in cancer cells, leading to the upregulation of the PI3K/AKT signaling pathway and cancer development. Overall, this basic research reveals the potential role of Prevotella and other specific lung cancer-associated bacteria in lung cancer development, providing a possible avenue for researching new preventive and therapeutic strategies (He et al., 2022). There is also a study that shows a potential association between Actinomyces and lung cancer development. The relative abundance of Actinomyces is increased in both COPD and NSCLC patients, and its abundance is positively correlated with the risk of developing lung cancer. This study reveals an association between the dysbiosis of lung microbiota and the occurrence of lung cancer. However, further research is necessary to determine the precise impact of Actinomyces on lung cancer development (Peters et al., 2022). It is worth noting that there is currently no research directly determining how the genus Ruminiclostridium 6 and Actinomycetales influence lung cancer. However, a study has shown that patients with a higher relative abundance of Actinomycetales have a lower risk of lung cancer recurrence after surgery (Wei et al., 2023). Further research is needed to determine the exact mechanism of action of Actinomycetales and other bacterial genera in lung cancer development.

It is worth noting that our research shows significant differences in results when compared to two other studies, those by Long et al. and Wei et al (Long et al., 2023; Wei et al., 2023), with the primary difference being that we used a larger and more comprehensive database. This makes our results more reliable, confirming previous research and providing a novel perspective on the potential causal relationship between GM and lung cancer. Additionally, we had stringent requirements for maintaining consistency between beta values and the IVW method in our analysis, which led to the exclusion of certain results due to slight differences. Additionally, unlike studies with limited sample sizes or specific populations, the use of MR studies has the advantage of overcoming confounding factors. MR uses natural genetic variation as instrumental variables to explore causality between the exposure and outcome. This approach can eliminate reverse causality and collinearity and exclude most confounding factors during the estimation process, leading to results that are more likely to reflect the true cause-effect relationship. However, there may still be some confounding factors present in the study, such as smoking, diet, medications, and COPD, which could interfere with the composition of the GM and increase the risk of developing lung cancer. As such, future research should continue to explore and implement advanced methods to reduce the influence of confounding factors.

This research has several advantages. First, we utilized MR analysis to minimize the effects of reverse causation and confounding, thus enhancing the causal inference of lung cancer and the GM. Second, we chose a more comprehensive and larger database, which yielded more results. Additionally, to confirm the robustness of our results, we also employed the MR-Egger regression intercept term test and MR-PRESSO. Nevertheless, this study still has some limitations. One limitation is that the molecular biology of the GM is not yet fully understood, which limits our ability to deduce the molecular mechanisms that link GM and lung cancer. Additionally, the lung cancer cases are primarily from cross-sectional studies, which may introduce a survival bias effect. At the same time, potential influential factors such as smoking, diet, or treatment effects may also affect the results of genome-wide association studies (GWAS). Finally, the majority of data in this study comes from European populations, and while we have included some other ethnic groups, our findings may be somewhat biased.

## 5 Conclusions

By conducting an MR analysis on the causal relationship between the GM and various subtypes of lung cancer, we identified potential causal associations between 10 microbial communities and lung cancer, 10 with lung adenocarcinoma, 9 with lung squamous cell carcinoma, and 11 with small cell lung cancer. Following correction, Peptococcaceae still displayed a strong causal relationship with lung adenocarcinoma. However, definitive experimental research is required to further explore the precise mechanism underlying this relationship.

## Data availability statement

The datasets presented in this study can be found in online repositories. The names of the repository/repositories and accession number(s) can be found in the article/**Supplementary Material**.

## Author contributions

MZ and YW contributed to the concept and design of this study. YL and KW were responsible for the statistical analysis and writing of the report. YZ and JY reviewed the article and provided critical feedback to improve and structure the report. YL and KW are regarded as co-first authors with the same degree of contribution. All authors contributed to the article and approved the submitted version.

## Supplementary material

Supplementary Figure 1 Scatter plots for the causal association between gut microbiota and lung cancer. MR, Mendelian randomization; SNP, single-nucleotide polymorphism.

Supplementary Figure 2 Scatter plots for the causal association between gut microbiota and lung adenocarcinoma. MR, Mendelian randomization; SNP, single-nucleotide polymorphism.

Supplementary Figure 3 Scatter plots for the causal association between gut microbiota and squamous cell lung cancer. MR, Mendelian randomization; SNP, single-nucleotide polymorphism.

Supplementary Figure 4 Scatter plots for the causal association between gut microbiota and small cell lung cancer. MR, Mendelian randomization; SNP, single-nucleotide polymorphism.

Supplementary Figure 5 Leave-one-out plots for the causal association between gut microbiota and lung cancer. MR, Mendelian randomization.

Supplementary Figure 6 Leave-one-out plots for the causal association between gut microbiota and lung adenocarcinoma. MR, Mendelian randomization.

Supplementary Figure 7 Leave-one-out plots for the causal association between gut microbiota and squamous cell lung cancer. MR, Mendelian randomization.

Supplementary Figure 8 Leave-one-out plots for the causal association between gut microbiota and small cell lung cancer. MR, Mendelian randomization.
