# Supplementary figures and images for: Revealing a causal relationship between gut microbiota and lung cancer: a Mendelian randomization study
Source: Front Cell Infect Microbiol. 2023 Sep 27;13:1200299. doi: 10.3389/fcimb.2023.1200299 (PMC10565354; doi:10.3389/fcimb.2023.1200299)

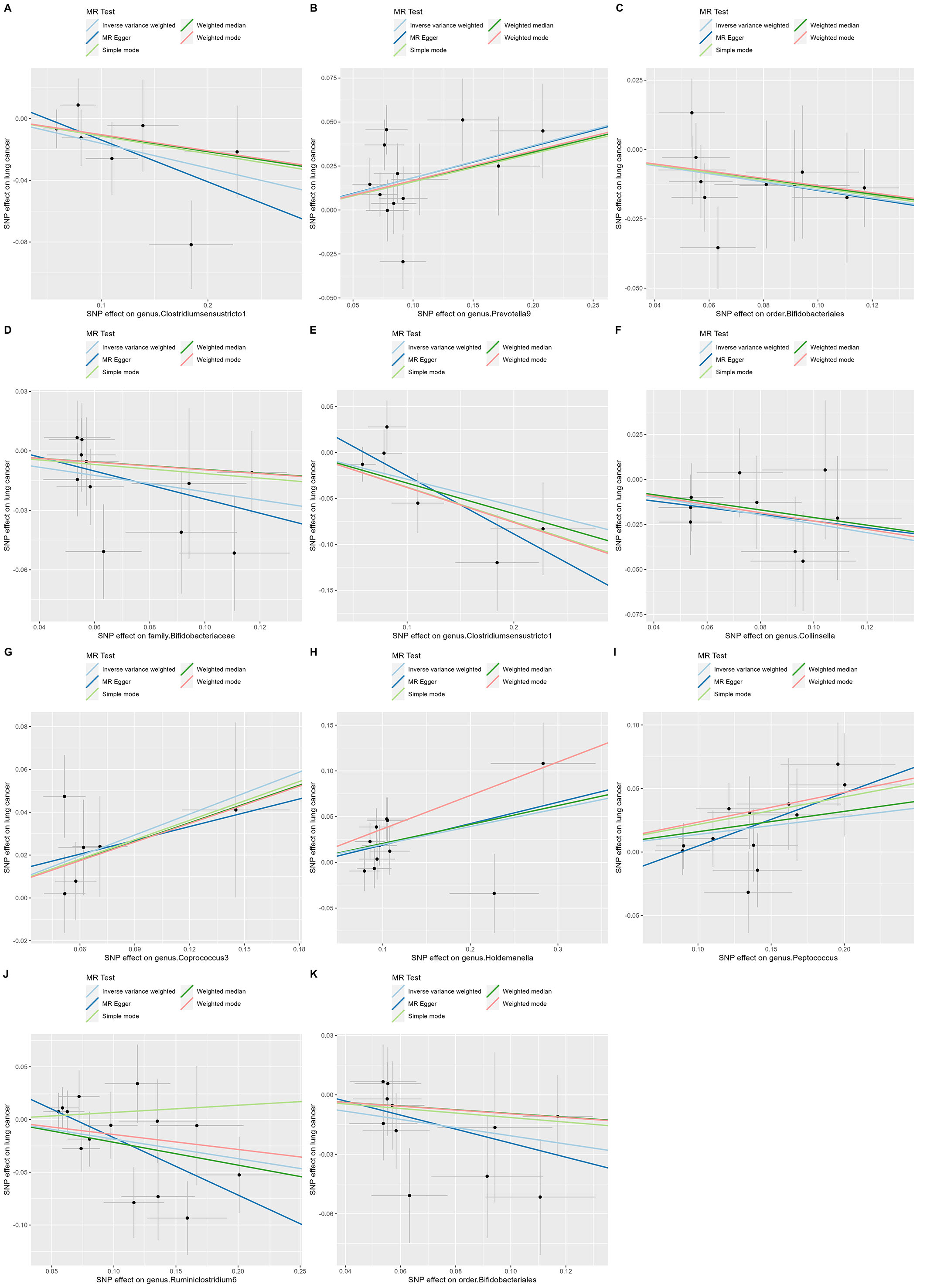

Supplement: Supplementary Figure 1 — Scatter plots for the causal association between gut microbiota and lung cancer. MR, Mendelian randomization; SNP, single-nucleotide polymorphism. [file DataSheet_1.zip › Supplementary Figure 1 in JPEG format.jpg]

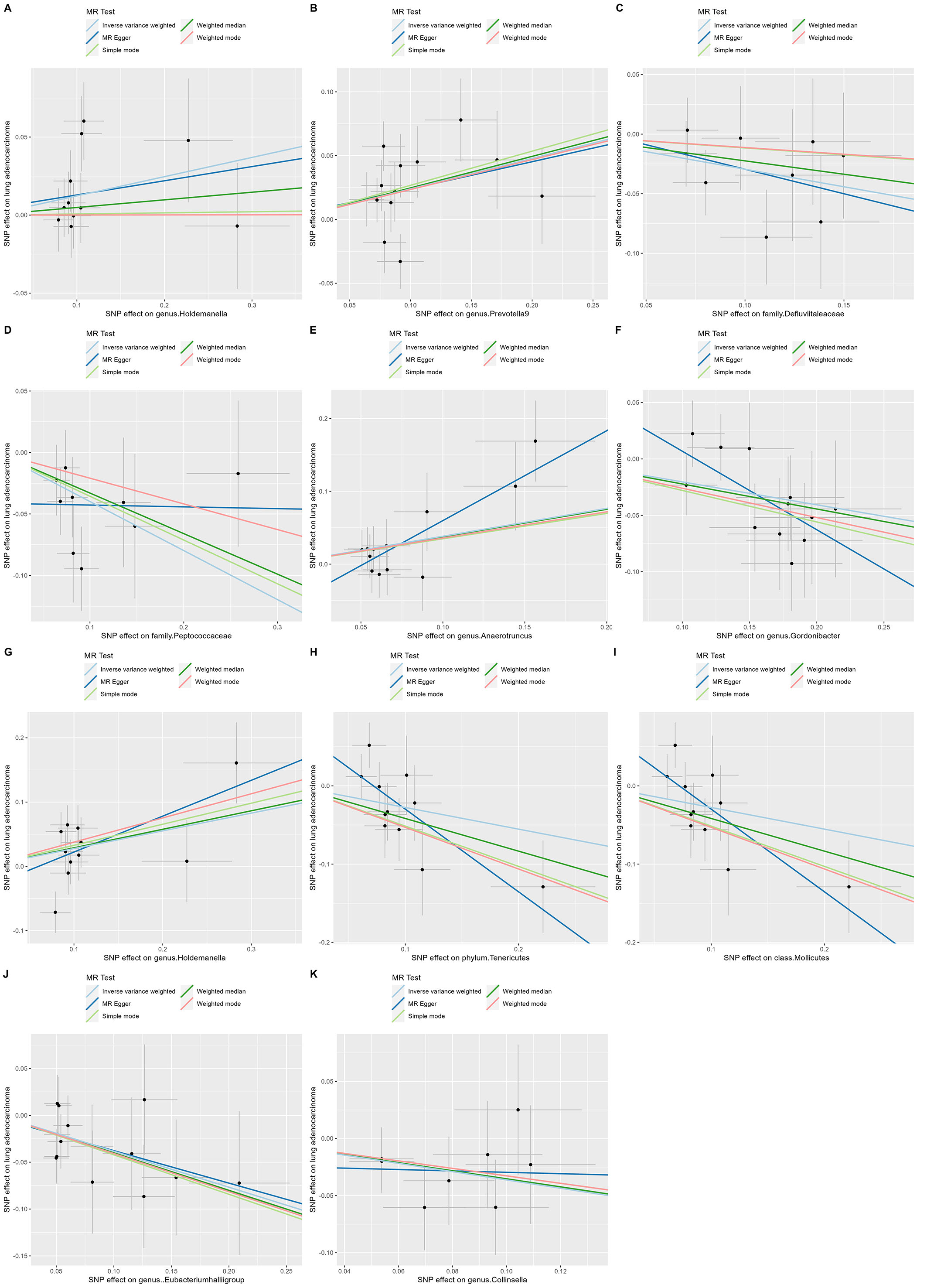

Supplement: Supplementary Figure 1 — Scatter plots for the causal association between gut microbiota and lung cancer. MR, Mendelian randomization; SNP, single-nucleotide polymorphism. [file DataSheet_1.zip › Supplementary Figure 2 in JPEG format.jpg]

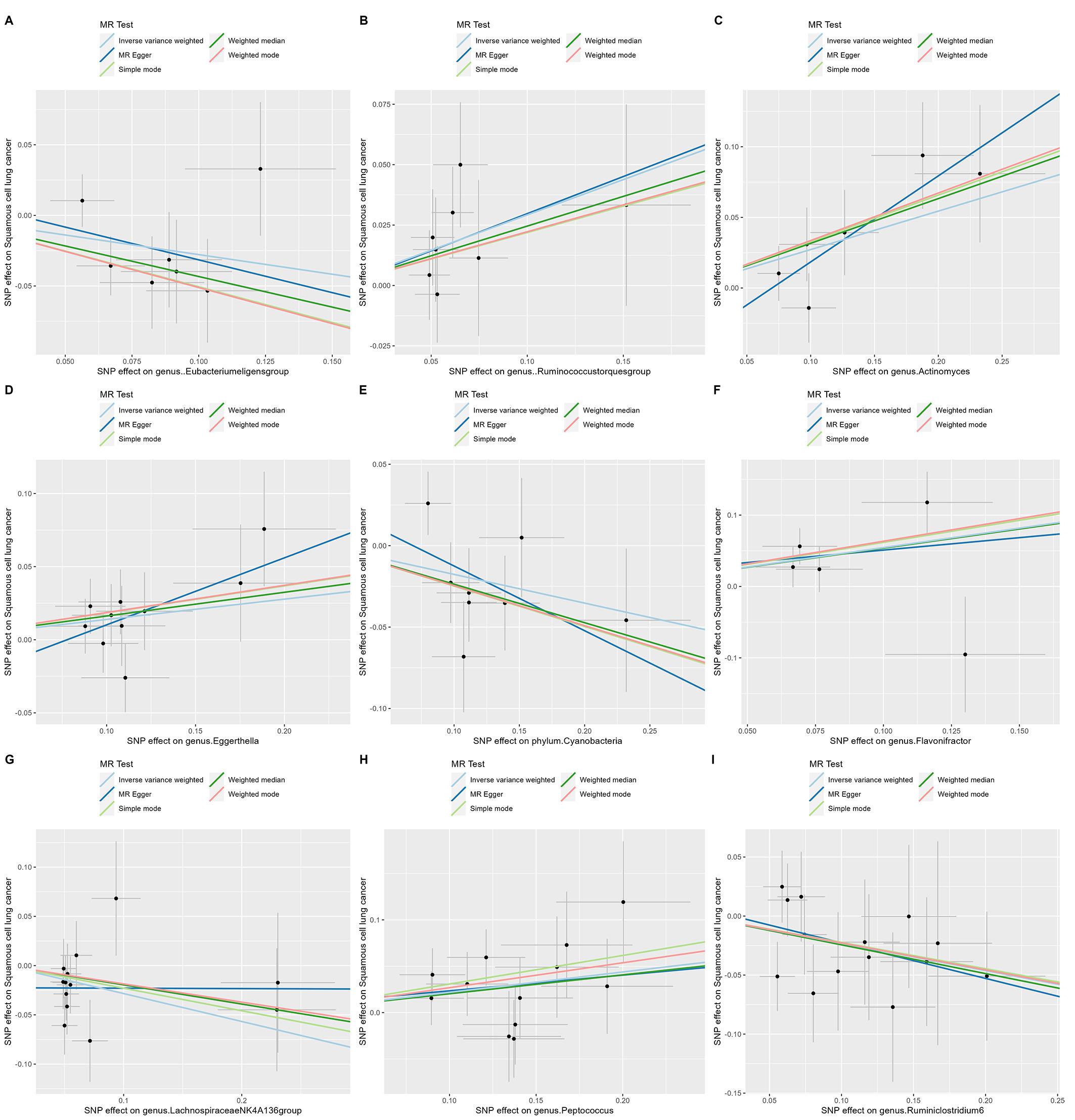

Supplement: Supplementary Figure 1 — Scatter plots for the causal association between gut microbiota and lung cancer. MR, Mendelian randomization; SNP, single-nucleotide polymorphism. [file DataSheet_1.zip › Supplementary Figure 3 in JPEG format.jpg]

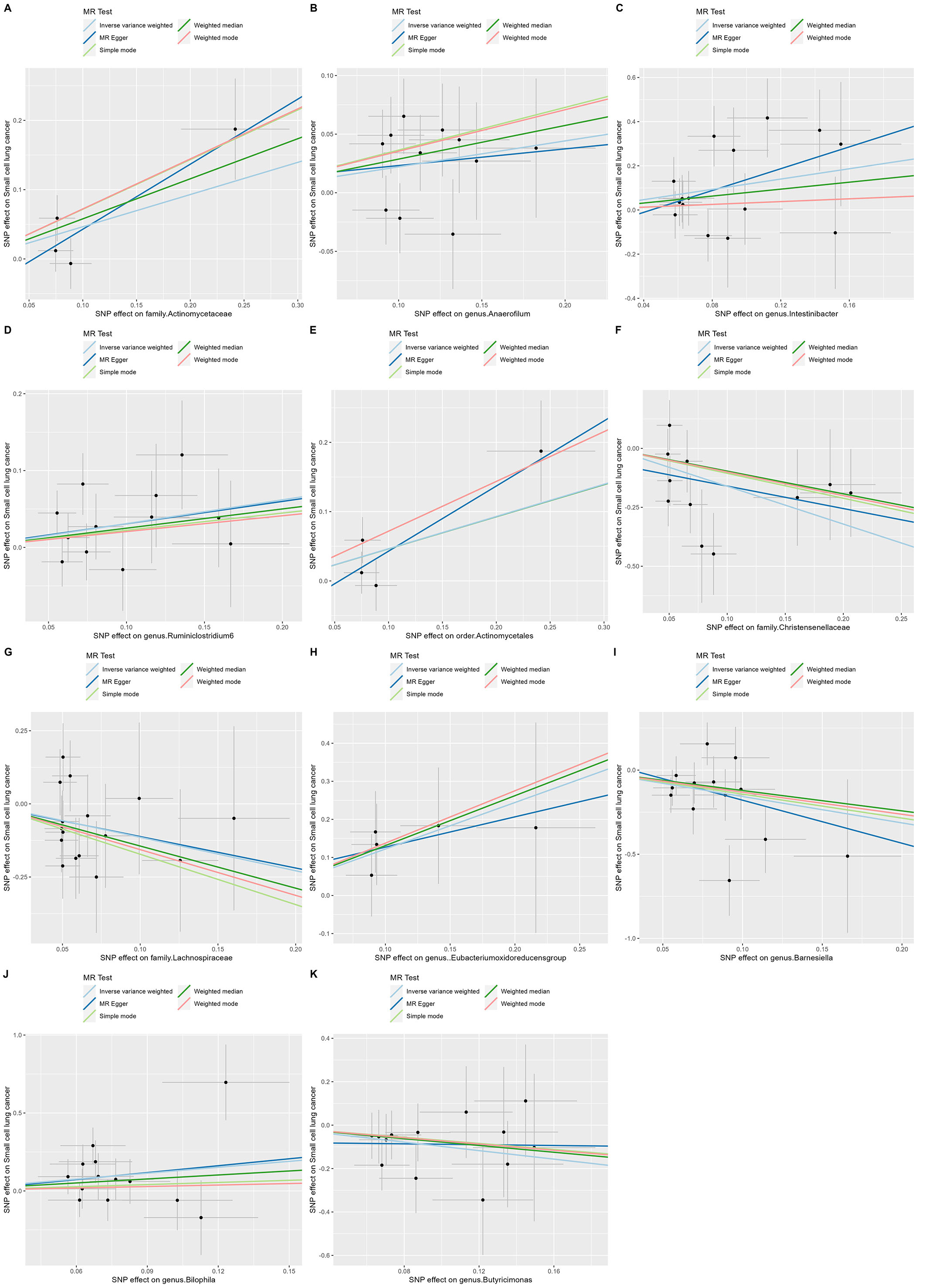

Supplement: Supplementary Figure 1 — Scatter plots for the causal association between gut microbiota and lung cancer. MR, Mendelian randomization; SNP, single-nucleotide polymorphism. [file DataSheet_1.zip › Supplementary Figure 4 in JPEG format.jpg]

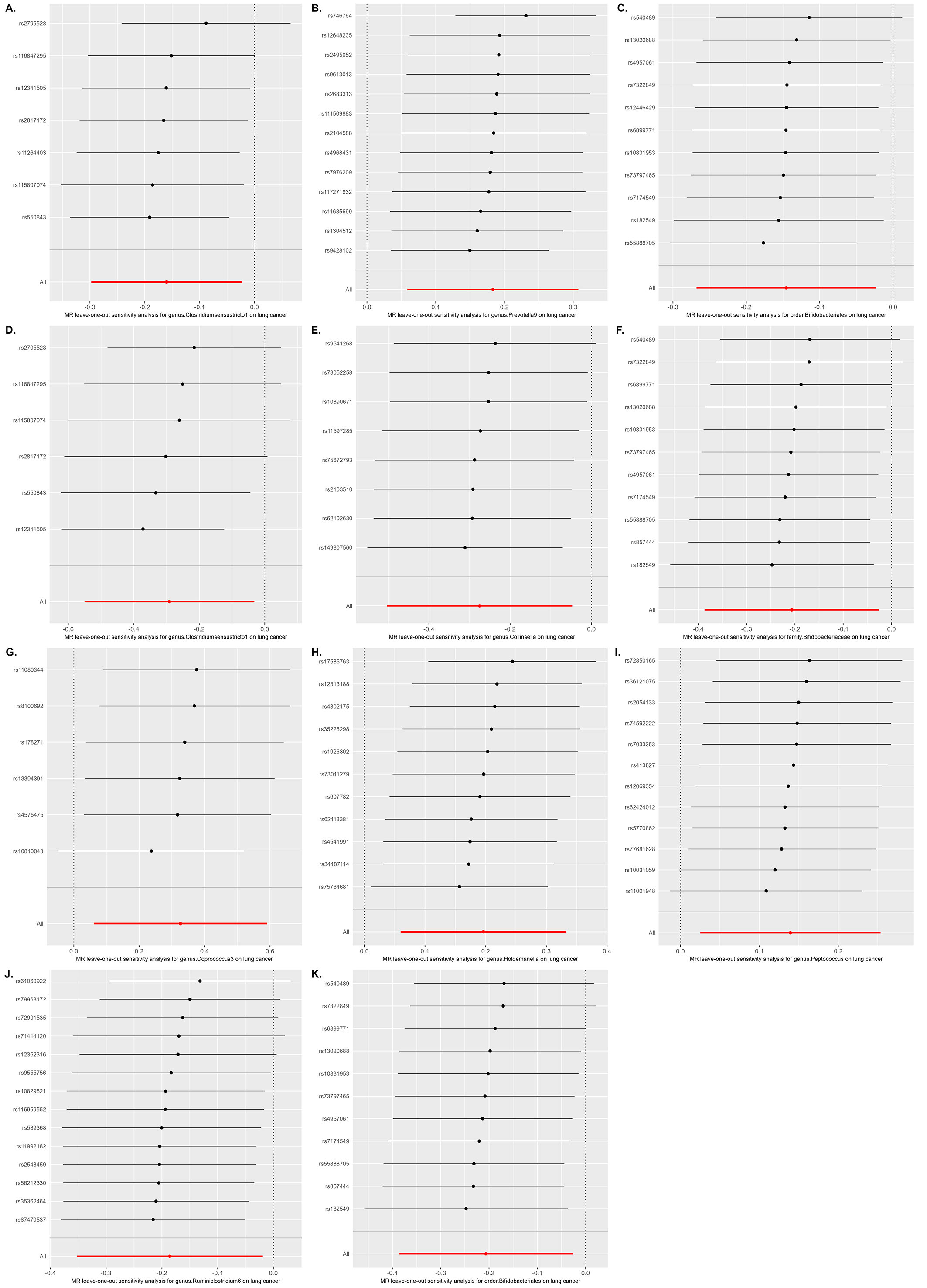

Supplement: Supplementary Figure 1 — Scatter plots for the causal association between gut microbiota and lung cancer. MR, Mendelian randomization; SNP, single-nucleotide polymorphism. [file DataSheet_1.zip › Supplementary Figure 5 in JPEG format.jpg]

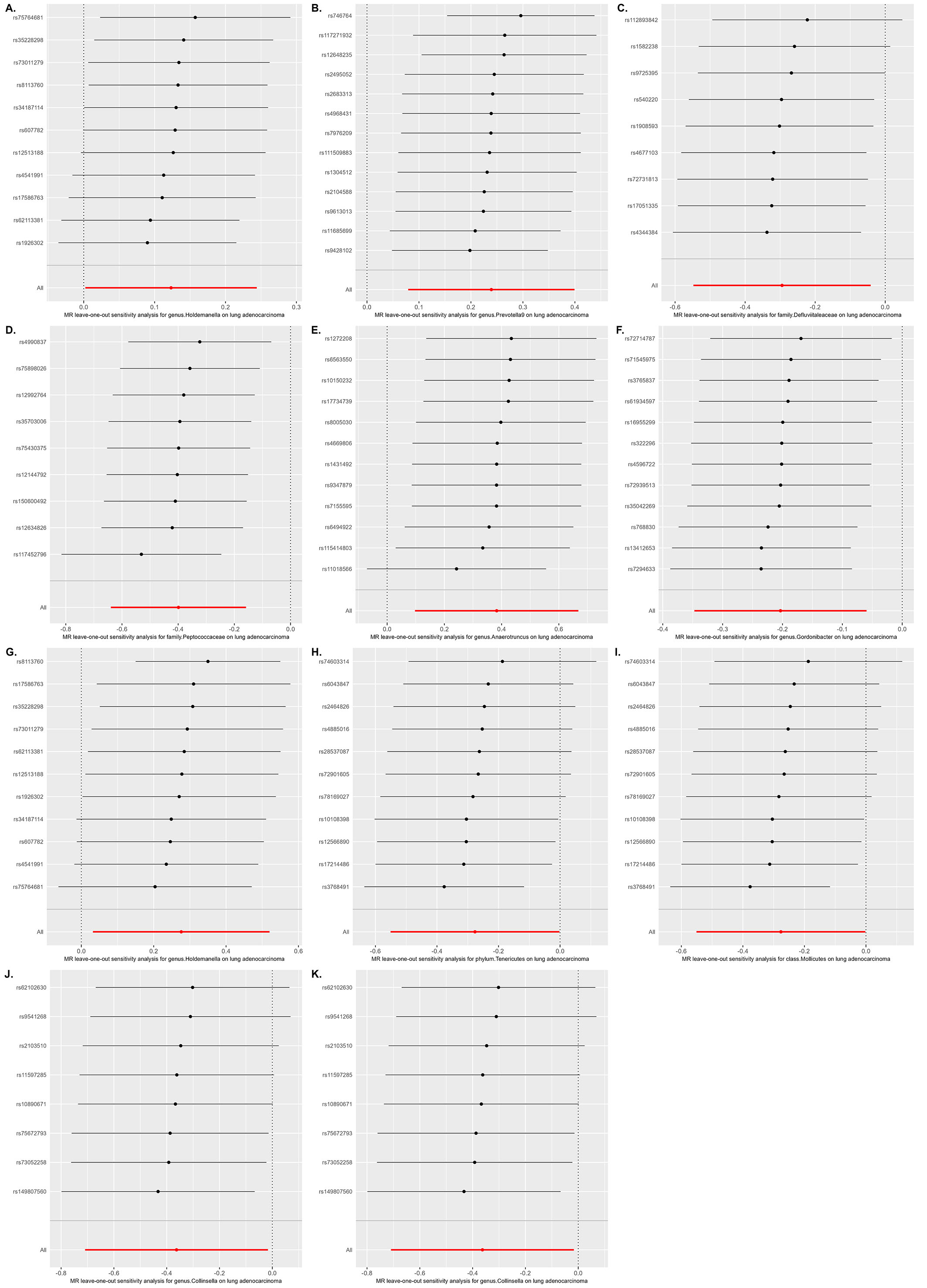

Supplement: Supplementary Figure 1 — Scatter plots for the causal association between gut microbiota and lung cancer. MR, Mendelian randomization; SNP, single-nucleotide polymorphism. [file DataSheet_1.zip › Supplementary Figure 6 in JPEG format.jpg]

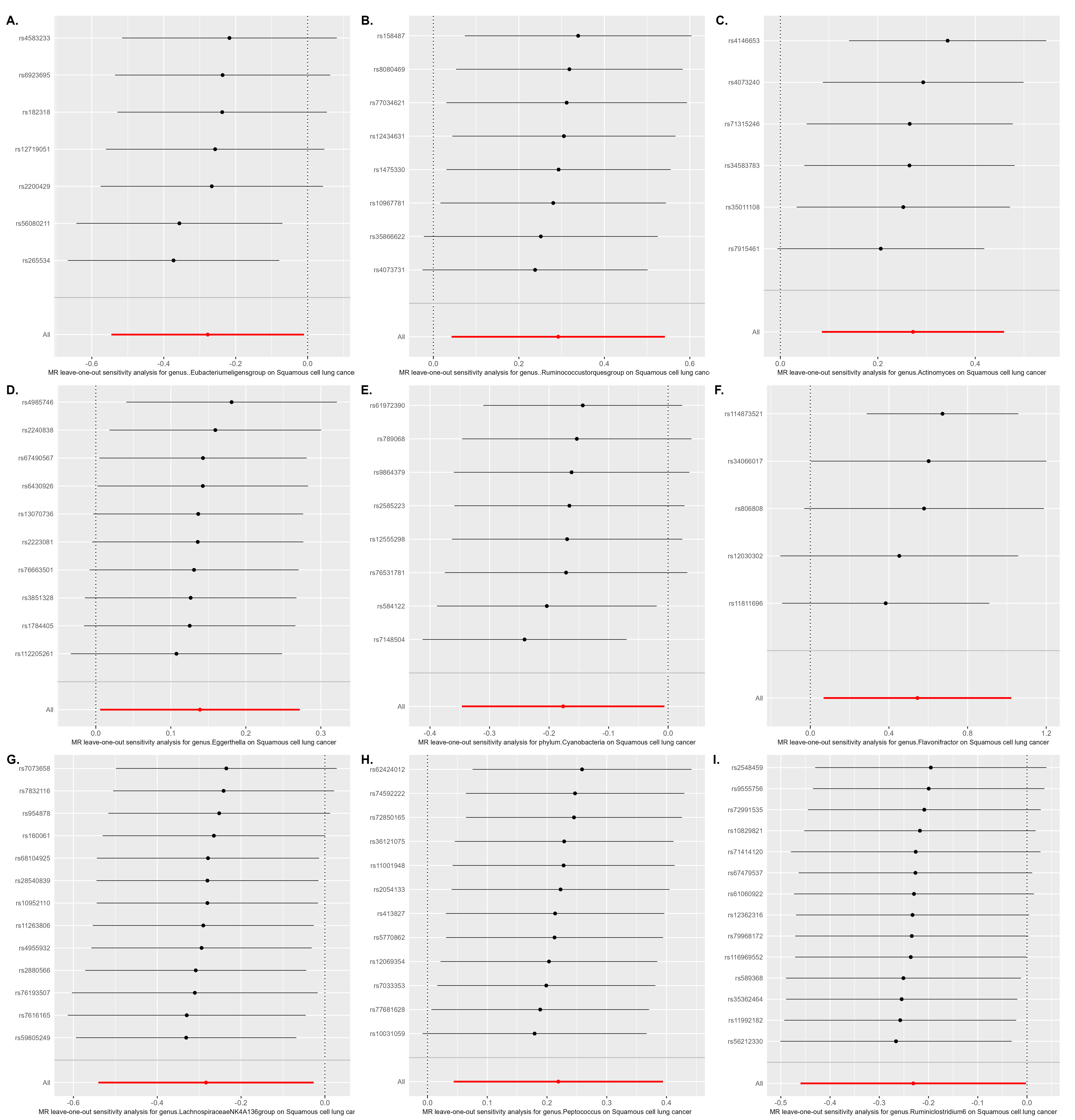

Supplement: Supplementary Figure 1 — Scatter plots for the causal association between gut microbiota and lung cancer. MR, Mendelian randomization; SNP, single-nucleotide polymorphism. [file DataSheet_1.zip › Supplementary Figure 7 in JPEG format.jpg]

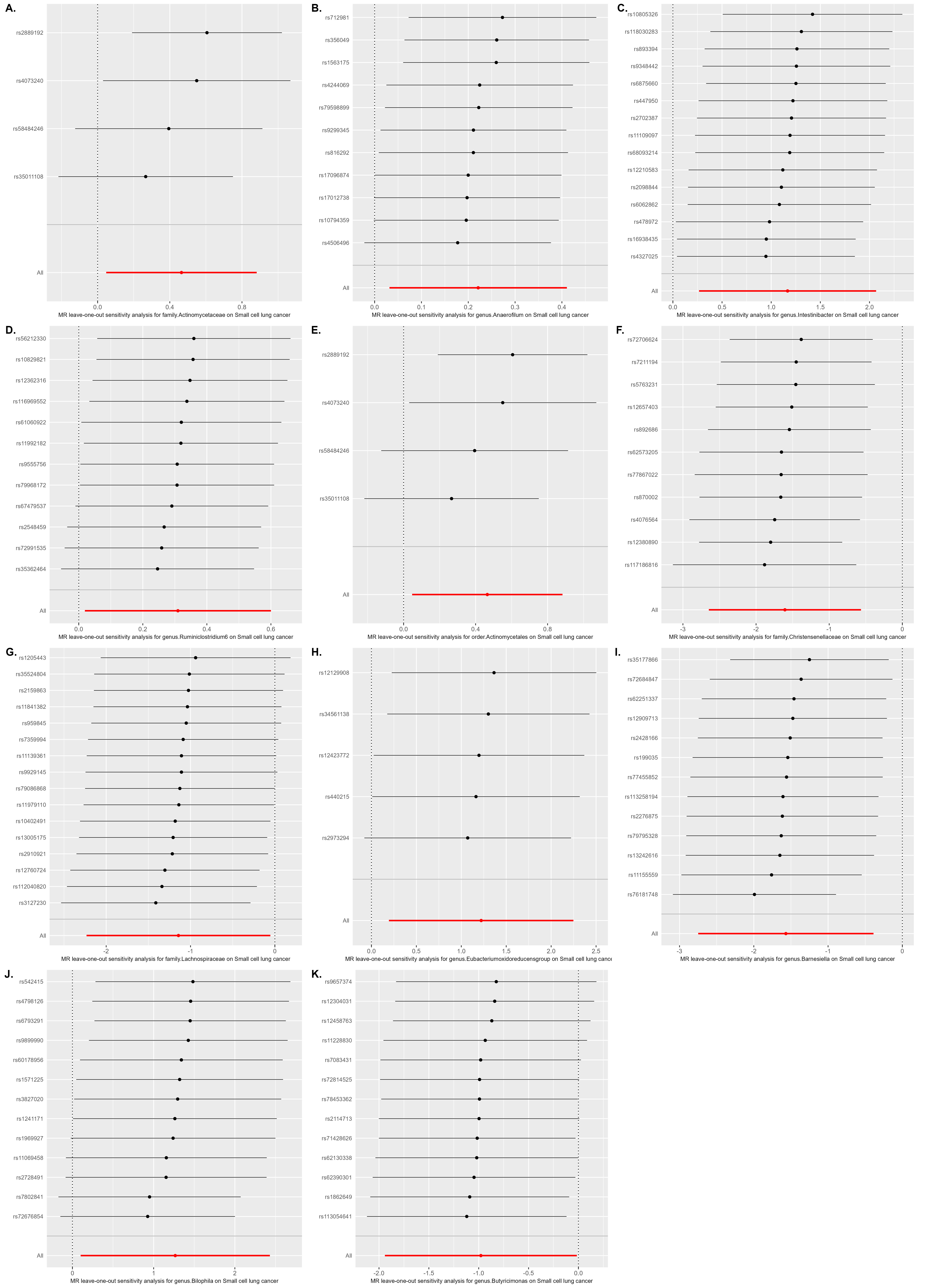

Supplement: Supplementary Figure 1 — Scatter plots for the causal association between gut microbiota and lung cancer. MR, Mendelian randomization; SNP, single-nucleotide polymorphism. [file DataSheet_1.zip › Supplementary Figure 8 in JPEG format.jpg]
